# Mean Platelet Volume as an Emerging Biomarker for Diagnosing Acute Appendicitis: A Retrospective Study

**DOI:** 10.7759/cureus.75247

**Published:** 2024-12-06

**Authors:** Shashirekha C.A., Akhil Vincent

**Affiliations:** 1 General Surgery, Sri Devaraj Urs Medical College, Kolar, IND

**Keywords:** appendicular abscess, appendicular inflammation, diagnosis of acute appendicitis, mean platelet volume(mpv), novel biomarker

## Abstract

Introduction

Acute appendicitis is a common surgical emergency that requires a timely and accurate diagnosis to prevent complications. Several laboratory markers have been assessed to improve the diagnostic accuracy of acute appendicitis, including C-reactive protein (CRP), white blood cell (WBC) count, and cytokines like interleukins and tumor necrosis factor-alpha. One less commonly used but potentially valuable marker is the mean platelet volume (MPV), which indicates the size of circulating platelets and has the potential to serve as a biomarker for inflammatory conditions.

Methodology

The study was designed as a retrospective analytical investigation to examine the role of MPV in diagnosing acute appendicitis. The retrospective design allowed the utilization of existing hospital data, ensuring that sufficient cases of acute appendicitis could be assessed without additional data collection.

The calculation was based on statistical parameters, including the standard deviations from previous studies, critical values at a 5% significance level, and an 80% test power, arriving at a minimum required sample size of approximately 100 to ensure adequate statistical power for meaningful conclusions.

Results

The study found that patients with acute appendicitis had significantly higher MPV compared to the normal reference value of 8.9 fL. The average MPV in the patient group was 10.25 fL, which was statistically higher than the reference value (*P* < 0.001).

Furthermore, the study revealed that MPV levels were even higher in patients with more severe forms of appendicitis, such as perforated or gangrenous appendicitis. The average MPV for the inflamed appendicitis group was 10.13 fL, while the average MPV for the perforated or gangrenous group was 11.26 fL, a statistically significant difference (*P* < 0.001).

Conclusions

The study demonstrates that MPV is a potentially valuable marker for diagnosing acute appendicitis and assessing its severity, particularly when used in combination with other biomarkers like neutrophil-to-lymphocyte ratio (NLR) and WBC count.

Elevated MPV in complicated cases of appendicitis, such as perforated or gangrenous appendicitis, suggests that MPV may aid in identifying high-risk patients who require urgent surgical intervention.

While MPV alone may not be as reliable as NLR in predicting appendicitis severity, its inclusion in a multi-biomarker approach could improve clinical decision-making.

## Introduction

Acute appendicitis is one of the most common surgical emergencies worldwide, with its high prevalence requiring timely and precise diagnosis to prevent complications [1]. Timely identification of acute appendicitis is essential to decrease morbidity and death, as delays in finding can lead to severe outcomes, including perforation and widespread infection [2]. Clinically, the condition presents with symptoms like sudden abdominal pain, nausea, and a notable migration of aches to the right iliac fossa, with indicative accuracy based on these clinical findings alone ranging between 70% and 80% [2]. However, since these symptoms are not always definitive and can overlap with other abdominal conditions, the need for improved diagnostic approaches is critical [3].

The pathophysiological mechanisms of acute appendicitis typically involve obstruction of the appendiceal lumen, often caused by a fecalith, lymphoid hyperplasia, or other blockages [1]. This obstruction leads to mucus buildup, increasing intraluminal and venous pressures, which subsequently causes ischemia in the appendiceal tissue. When luminal pressure exceeds approximately 85 mmHg, venules draining the appendix develop thrombosed, setting the stage for vascular congestion and engorgement, followed by the inflammation typical of acute appendicitis [1]. If left untreated, this ischemic condition can progress to gangrene and perforation, underscoring the importance of early and accurate diagnosis [4].

Several laboratory markers have been assessed to improve the diagnostic accuracy of acute appendicitis. These commonly include C-reactive protein (CRP), white blood cell (WBC) count, and various cytokines such as interleukins (IL-6 and IL-10) and tumor necrosis factor-alpha (TNF-α) [5,6]. Studies have demonstrated that elevated WBC and CRP can differentiate patients with appendicitis from those without, although these markers lack specificity for distinguishing between simple and complex cases of appendicitis [7]. One less commonly used but potentially valuable marker is the mean platelet volume (MPV), which indicates the size of circulating platelets. It can be easily measured in a routine complete blood count and has the potential to serve as a biomarker for inflammatory conditions, although it is often overlooked in the context of appendicitis diagnosis [5].

MPV is a measure of platelet activation, which can increase in response to inflammation. Elevated MPV levels are linked to various inflammatory diseases, and recent studies suggest that MPV may be altered in patients with acute appendicitis, potentially serving as a useful diagnostic marker [1,3]. The normal range for MPV in healthy individuals typically falls between 7.2 and 11.7 fL, with deviations possibly indicating the presence of inflammatory or thrombotic conditions [5]. While some research has explored the relationship between MPV and acute appendicitis, findings have been inconsistent, leading to a lack of consensus on MPV's diagnostic value for this condition. The use of MPV as a reliable indicator in this setting remains under-investigated, despite its accessibility and the minimal cost associated with obtaining this parameter [4,8].

Recent studies emphasize the need for alternative diagnostic markers that could complement current diagnostic methods for appendicitis. Research has demonstrated that MPV might be changed by cytokines such as IL-1 and IL-6, both of which are implied in inflammatory responses and could thereby contribute to platelet activation and size changes [6]. However, despite these promising associations, the clinical applicability of MPV in diagnosing appendicitis has yet to be firmly established [3]. For instance, a study by Salminen et al. questioned the consistency of MPV as a marker due to its variability across different patient populations and its association with other inflammatory conditions, which could potentially lead to diagnostic ambiguity in acute appendicitis cases [9]. The complexity of acute appendicitis also lies in its diverse presentation. While severe cases often necessitate immediate surgical intervention, particularly in instances of perforation or gangrene, some cases classified as uncomplicated might resolve with antibiotic treatment or even spontaneously [9,10]. This range in severity has led some researchers to propose that uncomplicated and complicated appendicitis represent distinct points on a spectrum of the disease rather than a single pathological process [10]. As such, the ability to distinguish between different severities of appendicitis would enhance clinical decision-making and potentially reduce unnecessary surgical interventions [7].

The lacuna in current knowledge regarding MPV as a diagnostic marker in appendicitis is highlighted by the inconsistent findings across studies. Although MPV has shown potential in small, retrospective studies, larger, more rigorous research is required to validate its diagnostic utility across diverse patient populations [8]. The lack of standardization in MPV measurement, along with its sensitivity to various confounding factors like platelet count variations and secondary health conditions, presents challenges that limit its current clinical use [5,6]. Thus, this study aims to further investigate MPV levels in patients with acute appendicitis and evaluate its potential as a diagnostic indicator, considering both uncomplicated and complicated cases.

This paper aims to analyze whether MPV can serve as a reliable biomarker for acute appendicitis, adding value to routine diagnostic practices. By analyzing MPV levels in a cohort of patients with confirmed appendicitis, this study seeks to determine whether significant differences in MPV exist between appendicitis cases and healthy reference values [3,4]. Furthermore, we aim to assess the diagnostic precision of MPV, considering its potential modulation by inflammatory cytokines like IL-1 and IL-6, which are involved in the inflammatory response associated with appendicitis [6]. In short, while MPV is an accessible and cost-effective parameter, its role in diagnosing acute appendicitis has yet to be fully validated. This study addresses this gap by examining MPV's diagnostic relevance and assessing its ability to differentiate between the severity levels of appendicitis. If MPV proves to be a reliable marker, it could become a valuable addition to the array of diagnostic tools available for appendicitis, especially in settings where rapid, accurate diagnosis is critical. Thus, this investigation seeks to add to the growing body of research on MPV and its potential to improve clinical outcomes in appendicitis cases.

## Materials and methods

Study design

This study was designed as a retrospective analytical investigation to examine the role of MPV in diagnosing acute appendicitis. The retrospective design allowed us to utilize existing hospital data, ensuring that sufficient cases of acute appendicitis could be assessed without additional data collection.

Sample size

The research included a sample of 100 patients. The sample size was determined using statistical parameters, including standard deviations from previous studies by Narci et al., critical values at a 5% significance level, and a test power of 80%. These factors indicated a minimum required sample size of approximately 100 to ensure sufficient statistical power for meaningful conclusions [1].

Inclusion and exclusion criteria

The inclusion criteria were established to ensure the relevance and consistency of the data. Only patients aged over 18 years with a pathology-confirmed diagnosis of acute appendicitis were included in the study. Exclusion criteria were applied to remove confounding variables related to chronic or unrelated health conditions. Patients with hematological diseases, cancer, chronic infections, or hepatic diseases were excluded from the study to avoid potential interference with MPV levels and ensure a more accurate analysis of MPV as a biomarker for acute appendicitis.

Data collection

The data for this study were retrospectively collected from the medical records of patients admitted to the surgical wards at R. L. Jallapa Hospital, all of whom had a confirmed diagnosis of acute appendicitis. The dataset included both demographic and clinical information relevant to evaluating MPV in the context of acute appendicitis. Demographic information included patients’ age and gender, while clinical data encompassed several indicators associated with the condition. These indicators included abdominal pain, nausea, vomiting, and fever, which are typical symptoms of acute appendicitis and provide insight into the clinical presentation. Additionally, WBC count and neutrophil count were recorded as routine hematological parameters to assess the systemic inflammatory response. The primary variable of interest, MPV, was documented as a routinely available hematological parameter, allowing for a comparison of MPV reference ranges between patients with acute appendicitis and healthy subjects. Operative notes were examined to provide detailed information on the severity of appendicitis, identifying cases as inflamed, gangrenous, or perforated. Histopathology reports were also reviewed to confirm the diagnosis for each case, thereby supporting accurate classification within the study.

Statistical analysis

A thorough statistical analysis was carried out to evaluate MPV as a potential diagnostic marker for acute appendicitis, comparing it to the reference MPV ranges in healthy individuals. The analysis incorporated descriptive statistics, detailing quantitative variables such as age, WBC count, and MPV, which were summarized by their minimum, maximum, mean, and standard deviation values, while categorical variables like gender and the type of appendicitis were visualized in charts to give an overview of the dataset. Inferential statistics were applied to compare MPV values between acute appendicitis patients and the normal reference range, utilizing a one-sample t-test to determine if MPV significantly deviated from healthy levels. This analysis employed a significance level of *P* < 0.05. To examine relationships between MPV and other clinical parameters, Pearson’s correlation coefficient was used, allowing assessment of correlations between MPV and markers of inflammation such as WBC and neutrophil count, providing insight into MPV’s pathophysiological relevance in acute appendicitis. Additionally, MPV levels were examined within subgroups of appendicitis severity (inflamed, gangrenous, or perforated), with an independent t-test used to identify significant differences across severity levels, which is crucial for evaluating MPV's role as an indicator of disease severity. Statistical procedures were performed using SPSS version 21.0 (IBM Corp., Armonk, NY), and the results were deemed statistically significant if the *P*-value was less than 0.05, ensuring a stringent examination of the relationship between MPV and acute appendicitis.

Ethical considerations

Given the retrospective type of this study, ethical consent was sought and obtained, with measures in place to ensure patient confidentiality. Data were anonymized before analysis, adhering to institutional guidelines on research ethics and patient privacy.

## Results

**Figure 1:**
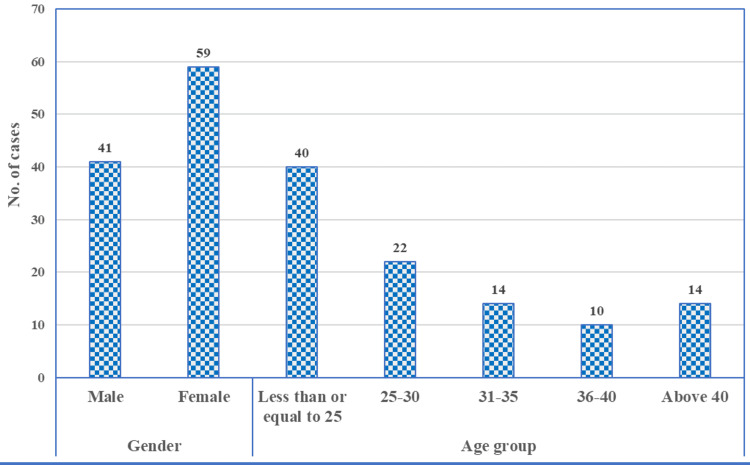
Distribution of patients based on demographic characteristics.

The results summarize the demographic information, clinical symptoms, and laboratory results of patients diagnosed with acute appendicitis. Patient ages ranged from 19 to 56 years, with an average age of 29.67 and a standard deviation of 9.12. The distribution of patients based on demographic characteristics shows a higher prevalence of acute appendicitis in females, with 59 female cases compared to 41 male cases (Figure 1). The age group most affected is individuals aged ≤25, with 40 cases, followed by the 25-30 age group, which accounts for 22 cases. The age groups 31-35 and above 40 had 14 cases, while the least affected age group was 36-40, with only 10 cases. This distribution suggests that younger adults, particularly those under 30, are more frequently diagnosed with acute appendicitis, with a slight female predominance.

**Figure 2:**
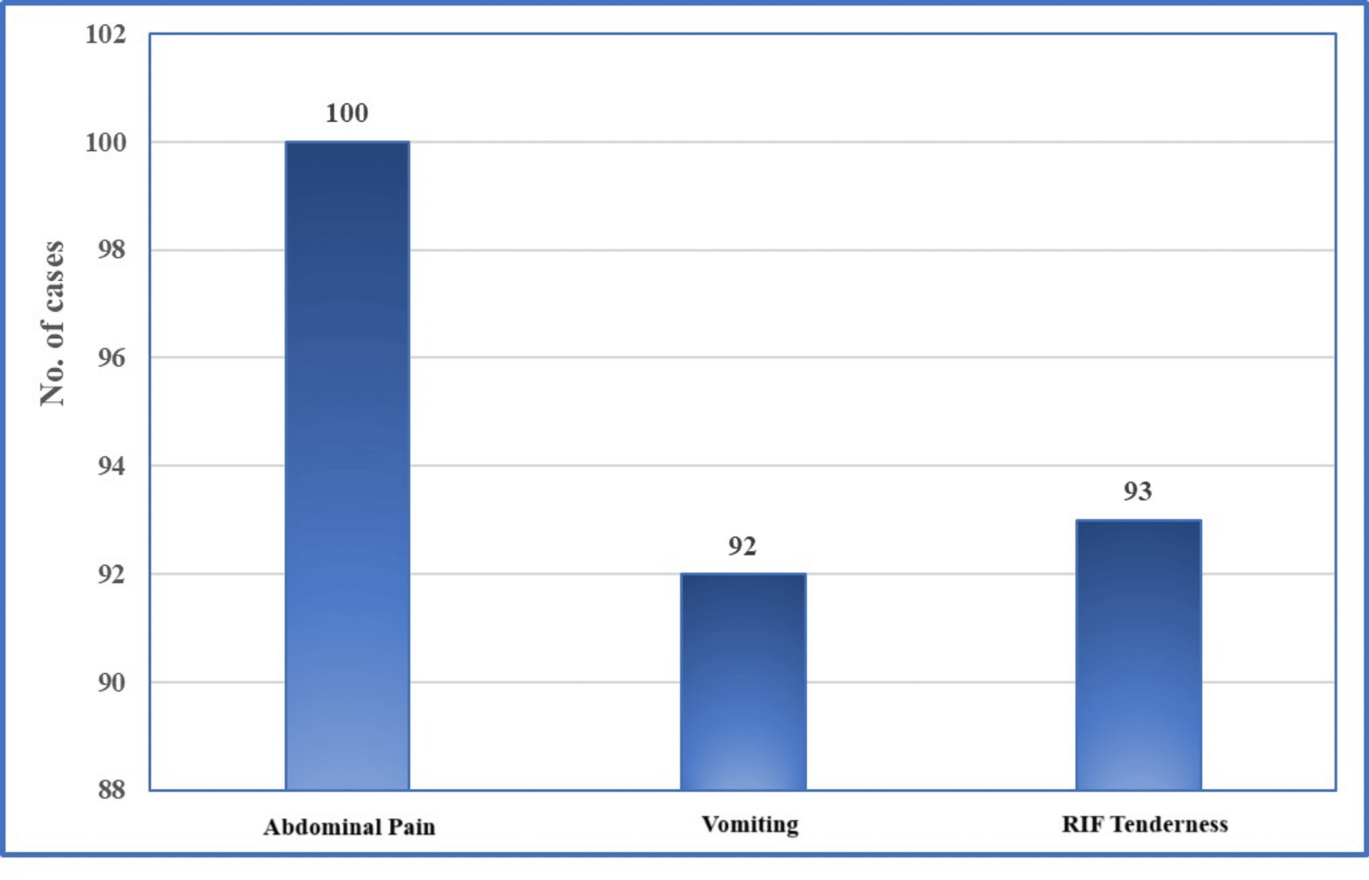
Clinical symptoms at the time of presentation at the hospital.

In Figure 2, clinical symptoms at the time of presentation highlight that abdominal pain was a universal symptom in all 100 cases. Other common symptoms included vomiting and tenderness in the right iliac fossa (RIF). Vomiting was stated in 92 cases, while RIF tenderness was noted in 93 cases. The high prevalence of these symptoms underscores their significance as key clinical indicators of acute appendicitis.

**Figure 3:**
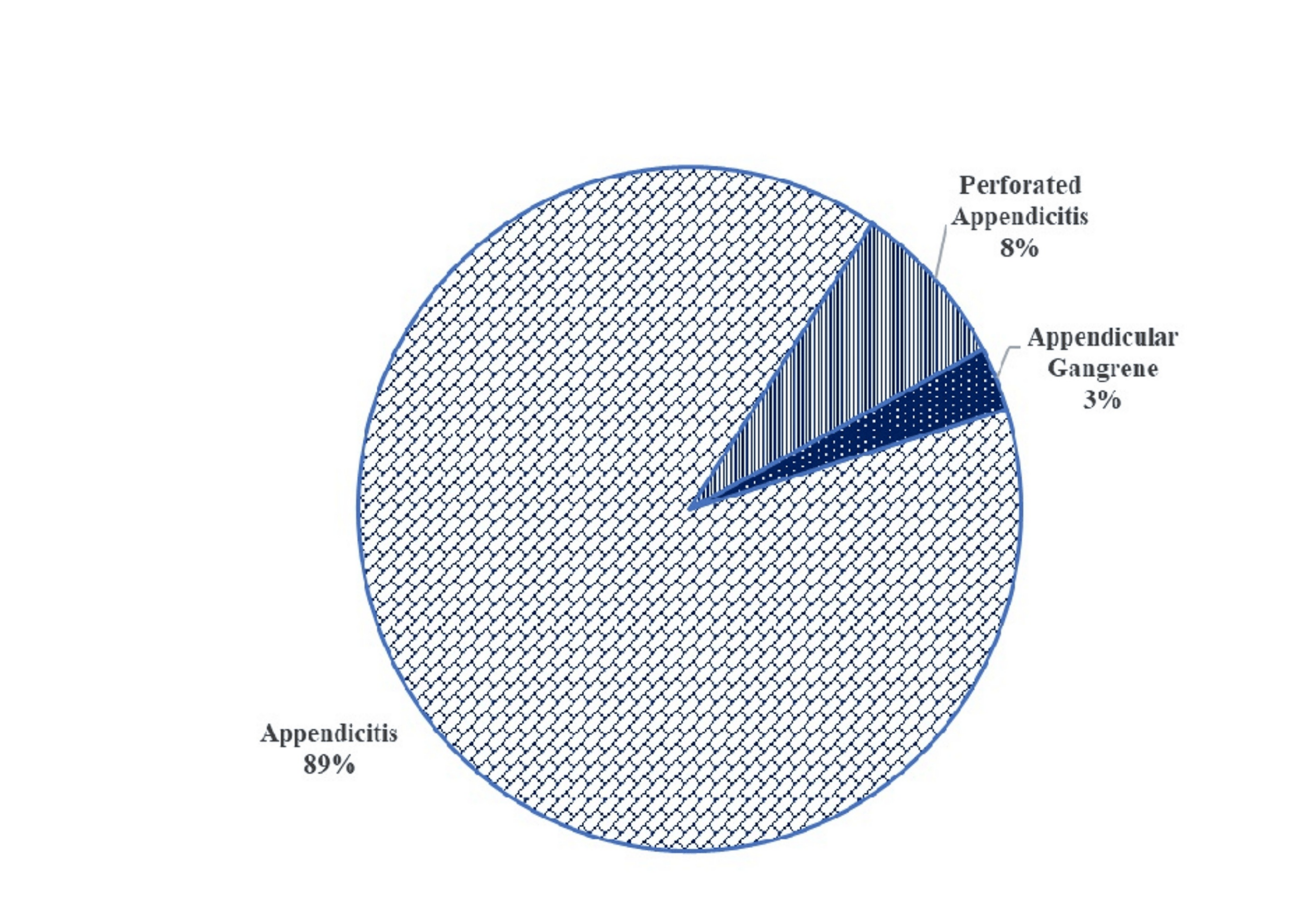
Classification of cases based on operative findings.

Based on operative findings (Figure 3), the majority of cases (89%) were diagnosed with uncomplicated appendicitis. Complicated cases, including perforated appendicitis, accounted for 8% of the total, while appendicular gangrene was observed in 3% of cases. This classification suggests that most patients presented with uncomplicated cases of appendicitis, but a small subset had more severe forms requiring urgent intervention.

The clinical data analysis indicates that the patients had an average age of 29.67 years, with a standard deviation of 9.12, and their ages ranged from 19 to 56 years (Table 1). Respiratory rate measurements showed considerable variability, with an average of 39.98 breaths per minute and a high standard deviation, suggesting the presence of outliers or measurement irregularities. Systolic blood pressure averaged 114.10 mmHg, while diastolic blood pressure averaged 76.50 mmHg. Heart rate values were unusually high, with a mean of 185.99 breaths per minute and a standard deviation of 920.06, suggesting possible errors in data recording. The mean body temperature was 38.33 °C, within the febrile range for appendicitis. Hemoglobin levels averaged 11.30 g/dL, while platelet count averaged 266.26 (thousands/cu.mm). The mean hematocrit was 37.29%, and serum creatinine averaged 0.87 mg/dL, indicating normal renal function. The mean white blood cell count was 17.42 (10^3/µL), with neutrophils averaging 14.41 (10^3/µL), which is consistent with an inflammatory response. The average lymphocyte count was 2.49 (10³/µL), and the average platelet volume was 10.25 fL, both of which are within the normal range.

**Table 1:** Descriptive statistics regarding clinical variables. Systolic BP, systolic blood pressure; diastolic BP, diastolic blood pressure; WBC, white blood cells; SD, standard deviation

Variables	Minimum	Maximum	Mean	SD
Age (Years)	19	56	29.67	9.12
Respiratory rate (breaths/minute)	16	2018	39.98	199.81
Systolic BP (mmHg)	80	130	114.10	12.07
Diastolic BP (mmHg)	60	90	76.50	7.96
Heart rate (breaths/minute)	72	9294	185.99	920.06
Temperature (degree Celsius)	37.2	39.3	38.33	0.48
Hemoglobin	7.8	16.8	11.30	1.65
Platelets (thousands/cu.mm)	144	896	266.26	81.71
Hematocrit	23.20	45.60	37.29	5.88
Serum creatinine (mg/dL)	0.00	1.70	0.87	0.25
WBC (10^3^/µL)	13.25	24.36	17.42	2.07
Neutrophil (10^3^/µL)	10.55	21.44	14.41	1.96
Lymphocyte (10^3^/µL)	1.07	4.81	2.49	0.84
Mean platelet volume (fL)	9.10	12.00	10.25	0.52

In Table 2, the correlation analysis between MPV and various clinical variables reveals a few significant relationships. MPV exhibits a moderate inverse relationship with systolic blood pressure (*r* = -0.302, *P* < 0.01) and diastolic blood pressure (*r* = -0.344, *P* < 0.01), suggesting that as blood pressure declines, MPV levels tend to rise. Furthermore, MPV shows a slight negative correlation with hemoglobin levels (*r* = -0.207, *P* < 0.05), suggesting that patients with lower hemoglobin may have a slightly higher MPV. There is also a minor positive correlation between MPV and serum creatinine (*r* = 0.198, *P* < 0.05), which could imply a relationship between MPV and renal function in this group of patients. Other variables, such as age, respiratory rate, temperature, WBC count, and neutrophil count, do not show statistically significant correlations with MPV.

**Table 2:** Correlation of MPV with other clinical variables. MPV, mean platelet volume; systolic BP, systolic blood pressure; diastolic BP, diastolic blood pressure; WBC, white blood cells

Variables	Correlation
Age (Years)	0.020
Respiratory rate (Breaths/minute)	0.013
Systolic BP (mmHg)	-0.302
Diastolic BP (mmHg)	-0.344
Heart rate (Breaths/minute)	0.188
Temperature (Degree Celsius)	0.084
Hemoglobin	-0.207
Platelets (thousands/cu.mm)	0.027
Hematocrit	-0.173
Serum creatinine (mg/dL)	0.198
WBC (10^3^/µL)	0.064
Neutrophil (10^3^/µL)	0.033
Lymphocyte (10^3^/µL)	0.078

In Table 3, a comparison of MPV in the study group with the standard reference value of 8.9 fL reveals a statistically significant difference. The average MPV in the patient group was 10.25 fL, with a standard deviation of 0.52, which is notably higher than the reference value. The t-test analysis produced a *t*-value of 25.923 with a *P*-value less than 0.001, indicating a highly significant difference (*P* < 0.01). This suggests that individuals with acute appendicitis generally exhibit elevated MPV levels, highlighting the diagnostic importance of MPV in this condition.

**Table 3:** Comparison of mean platelet volume with normal value. *Significant at 0.01 level. SD, standard deviation

Variable	Mean	SD	Reference value	*t*-value	*P*-value
Mean platelet volume (fL)	10.25	0.52	8.9	25.923*	<0.001

Using a one-sample t-test, the MPV in the study group was compared to the normal reference value of 8.9 fL. The results indicate that the study group has a significantly higher MPV, with a mean of 10.25 fL (*P* < 0.001). This finding highlights a notable elevation in MPV among patients with acute appendicitis, suggesting that MPV could serve as a useful diagnostic marker to distinguish these patients from healthy individuals.

In Table 4, a comparison of MPV levels between patients with inflamed appendicitis and those with perforated or gangrenous appendicitis demonstrates a significant difference in MPV. The average MPV for the inflamed group (*n *= 89) was 10.13 fL, with a standard deviation of 0.36, while the average MPV for the perforated or gangrenous group (*n *= 11) was considerably higher at 11.26 fL, with a standard deviation of 0.54. The t-test analysis produced a *t*-value of 9.347 and a highly significant *P*-value of <0.001, indicating that the difference in MPV between the two groups is statistically significant (*P* < 0.01).

**Table 4:** Comparison of the mean platelet volume between the inflamed appendicitis group and the perforated or gangrene group. *Significant at 0.01 level. SD, standard deviation

Type of appendicitis	Mean	SD	*t*-value	*P*-value
Inflamed (*n *= 89)	10.13	0.36	9.347*	<0.001
Perforated or gangrene (*n *=11)	11.26	0.54		

These results indicate that MPV levels are significantly higher in patients with more severe forms of appendicitis, such as perforated or gangrenous appendicitis, compared to those with only inflamed appendicitis. This significant increase in MPV in complicated cases could indicate that MPV is not only a potential diagnostic marker for acute appendicitis but may also serve as an indicator of disease severity. Elevated MPV levels in the perforated or gangrenous group could reflect a heightened inflammatory or thrombotic response associated with advanced stages of appendicitis. As a result, these findings highlight the potential of MPV as a clinical biomarker to distinguish between uncomplicated and complicated appendicitis, which could assist in the timely management and prioritization of patients for surgical intervention.

## Discussion

The study found that acute appendicitis predominantly affects younger adults, with a slight female predominance, which is consistent with existing literature [11]. The mean age of patients in this study (29.67 years) aligns closely with findings from previous studies, indicating that appendicitis is common in individuals aged 20-40 [12]. Our study's demographic findings are also supported by Ishizuka et al., who noted a higher frequency of appendicitis in younger populations [13]. The universal presence of abdominal pain and high prevalence of vomiting and right iliac fossa tenderness among patients highlight the significance of these symptoms as primary indicators of appendicitis, corroborating the established diagnostic criteria [3].

In terms of clinical markers, this study reported elevated MPV levels among patients with acute appendicitis. With a mean MPV of 10.25 fL, which is significantly above the normal reference value of 8.9 fL, the results are in agreement with other studies that support MPV as an inflammatory marker in acute appendicitis [14]. However, some studies, such as Tuncer et al., have shown conflicting findings, reporting a decrease in MPV among appendicitis cases compared to controls [15]. This discrepancy may be attributed to differences in patient demographics, severity of appendicitis, and methodology in MPV measurement.

The findings have several clinical implications. The elevated MPV in patients with acute appendicitis, particularly in complicated cases like perforated or gangrenous appendicitis, suggests that MPV could serve as a valuable marker not only for diagnosis but also for assessing disease severity. Higher MPV levels in cases of complicated appendicitis, as observed in our study, align with the work of Aydogan et al. and Hajibandeh et al., who propose that elevated MPV reflects increased platelet activation and turnover due to systemic inflammation [11,16]. Additionally, the study found that MPV is inversely correlated with blood pressure and hemoglobin levels. A moderate negative correlation between MPV and systolic and diastolic blood pressure suggests a possible association between MPV and cardiovascular stress in acute appendicitis cases. This relationship calls for further study, as emphasized by Tullavardhana et al., to better understand how MPV may play a role in managing hemodynamic stability in patients with appendicitis [14]. Additionally, when combined with NLR and WBC count, MPV could improve diagnostic accuracy and assist in the early identification of complicated appendicitis, helping clinicians prioritize surgical interventions for higher-risk cases. Hajibandeh et al. demonstrated that NLR > 8.8 could distinguish complicated from uncomplicated appendicitis with high sensitivity and specificity, supporting the potential for a combined marker approach [16].

When compared to other biomarkers like NLR and WBC count, MPV offers both advantages and limitations. While WBC and NLR are established markers for inflammation, MPV adds value by providing insight into platelet activation, which is associated with vascular and systemic inflammatory responses [3,7]. The current study’s findings reinforce the role of MPV in detecting inflammation severity, especially in perforated or gangrenous appendicitis cases. Nevertheless, NLR seems to offer superior sensitivity and specificity in distinguishing uncomplicated from complicated appendicitis [16]. In our study, MPV was effective in differentiating between inflamed and complicated appendicitis, but previous research suggests that NLR values greater than 8 are more predictive of gangrenous cases [13]. Supangat et al. also found that while MPV and platelet distribution width (PDW) are valuable, NLR remains superior due to its direct correlation with the inflammatory burden [12]. This comparative analysis suggests that combining MPV with NLR and WBC count could improve the diagnostic precision for appendicitis severity. CRP, another commonly used marker, was not measured in this study but has been frequently associated with inflammation in appendicitis, particularly in combination with WBC [7,15]. Further research might explore the combined use of CRP, MPV, and NLR to develop a comprehensive biomarker profile for appendicitis diagnosis and severity assessment.

The elevation in MPV in patients with acute appendicitis can be attributed to systemic inflammatory response and increased platelet activation. Thrombopoiesis, the process of platelet formation, accelerates under inflammatory conditions, leading to the release of larger, immature platelets into circulation. These larger platelets are more metabolically active and have greater pro-inflammatory potential, which explains the elevated MPV observed in cases of acute inflammation like appendicitis [11,14]. In cases of complicated appendicitis, such as perforation or gangrene, the inflammatory response is more intense, resulting in significantly higher MPV values. Ishizuka et al. and Hajibandeh et al. propose that cytokines, especially IL-6, play a critical role in modulating platelet production and activation during severe inflammation [13,16]. These cytokines drive megakaryocyte proliferation and stimulate the release of young, larger platelets, contributing to elevated MPV levels. This physiological response is supported by the finding of a positive correlation between MPV and serum creatinine in this study, suggesting a possible link between MPV and renal function in the context of systemic inflammation. Elevated MPV in the context of acute appendicitis reflects the body’s compensatory response to inflammation, which also involves increased platelet turnover and vascular permeability [3,17]. Additionally, the negative correlation between MPV and hemoglobin could indicate a relationship between anemia and inflammatory burden, possibly due to the sequestration and utilization of red blood cells in inflamed tissue [15,18].

 Limitations

The limitations of this study highlight several factors that may influence the interpretation and generalizability of the results. First, the retrospective design of the study introduces inherent limitations in data accuracy and reliability. Retrospective analyses depend on previously collected data, which may lack the precision and control of prospective studies, potentially leading to biases or missing information that could affect outcome accuracy. Another limitation is the sample size of the study, which is a significant factor. A smaller sample size could reduce the statistical power, making it harder to apply the findings to a larger population. This restricts the study’s external validity, as the conclusions drawn may not be fully applicable to diverse patient populations or clinical settings. Furthermore, the presence of potential confounding variables must be acknowledged. Factors such as undiagnosed medical conditions or inconsistencies in laboratory techniques could have influenced MPV levels, potentially skewing the results. For instance, variations in equipment calibration or procedural methods across different laboratories might introduce variability in MPV measurements, impacting the study's internal validity. Addressing these limitations through more rigorous study designs and larger, controlled sample sizes in future research is essential for more accurate and generalizable findings.

## Conclusions

The study demonstrates that MPV is a potentially valuable marker for diagnosing acute appendicitis and assessing its severity, particularly when used in combination with other biomarkers like NLR and WBC count. Elevated MPV in complicated cases suggests that it may aid in identifying high-risk patients who require urgent surgical intervention. While MPV alone may not be as reliable as NLR in predicting appendicitis severity, its inclusion in a multi-biomarker approach could improve clinical decision-making. Further research is warranted to validate MPV’s role alongside other inflammatory markers and to explore its pathophysiological connections with systemic inflammation in appendicitis. Studies with larger sample sizes and diverse populations could help confirm these findings and establish standardized cut-off values for MPV in diagnosing and staging appendicitis severity.
